# Diagnostic value of congenital hypertrophy of the retinal pigment epithelium in familial adenomatous polyposis: a systematic review

**DOI:** 10.1186/s40942-026-00886-3

**Published:** 2026-06-23

**Authors:** Esmaeil Asadi Khameneh, Romina Dadkhah, Moein Zangiabadian, Amirreza Mafi, Amirhossein Hashemi, Mohamad Sanei, Ahmad Mirshahi, Zahra Mahdizad, Fatemeh Bazvand

**Affiliations:** Farabi Eye Hospital, Tehran University of Medical Sciences, Tehran, Iran

**Keywords:** Congenital hypertrophy of the retinal pigment epithelium, Familial adenomatous polyposis, Adenomatous polyposis coli gene

## Abstract

**Background:**

Congenital hypertrophy of the retinal pigment epithelium (CHRPE) has been proposed as a phenotypic marker of familial adenomatous polyposis (FAP). This systematic review aimed to evaluate the evidence regarding the association between CHRPE and FAP and to determine the potential clinical utility of CHRPE for the early identification of individuals with adenomatous polyposis coli (APC) mutations.

**Methods:**

A systematic search of PubMed/MEDLINE, Embase, and Web of Science from inception to August 2025 in accordance with PRISMA guideline and a PROSPERO-registered protocol was done. Studies reporting CHRPE in individuals with confirmed or suspected FAP or at-risk first-degree relatives were identified. Data extraction and quality assessment using Quality Assessment of Diagnostic Accuracy Studies-2 (QUADAS-2) tool were performed independently by two reviewers. Due to substantial heterogeneity in CHRPE definitions, diagnostic criteria, population characteristics, and APC mutation testing methods, quantitative pooling was not considered appropriate and findings were synthesized qualitatively.

**Results:**

Forty-three studies were included. CHRPE demonstrated consistently high specificity for FAP and APC mutation carriage across most studies. Sensitivity ranged from 40% to 100% in affected individuals and from 57% to 100% in at-risk relatives, while specificity ranged from 58% to 100%, depending on diagnostic criteria. Stricter definitions incorporating lesion multiplicity and bilaterality improved specificity at the expense of sensitivity.

**Conclusion:**

CHRPE is associated with FAP but demonstrates variable diagnostic performance across studies. Its clinical utility as a standalone screening marker is limited by heterogeneous diagnostic definitions. Standardized diagnostic criteria and prospective studies using contemporary genetic testing are needed.

**Supplementary Information:**

The online version contains supplementary material available at https://doi.org/10.1186/s40942-026-00886-3.

## Introduction

Familial adenomatous polyposis (FAP) is an autosomal dominant cancer predisposition syndrome caused by pathogenic variants in the adenomatous polyposis coli (APC) gene [1]. Affected individuals develop hundreds to thousands of colorectal adenomas and, in the absence of surveillance and prophylactic intervention, face an almost inevitable progression to colorectal carcinoma [2, 3]. Early identification of mutation carriers remains central to reducing morbidity and mortality, particularly among asymptomatic at-risk relatives [4]. 

Although molecular genetic testing is considered the diagnostic gold standard, its universal implementation remains constrained in certain settings by cost, accessibility, and variability in panel completeness [5]. Moreover, historical cohorts were evaluated before routine APC genotyping, complicating interpretation of older phenotypic studies. For these reasons, extracolonic manifestations of FAP have long been investigated as potential clinical markers of mutation carriage [1, 6, 7]. 

Congenital hypertrophy of the retinal pigment epithelium (CHRPE) is among the most recognized ocular findings associated with FAP [5, 8–11]. Multiple, bilateral, or morphologically characteristic lesions have been proposed as phenotypic indicators of APC mutations [2, 12–14]. However, reported diagnostic performance varies substantially across studies, reflecting heterogeneity in lesion definitions, ophthalmic examination techniques, study populations, and genetic confirmation methods [6, 10, 15–19]. 

Previous reviews have summarized the association between CHRPE and FAP, but most have not systematically accounted for differences in molecular confirmation, diagnostic thresholds for CHRPE, or variability in study design when interpreting diagnostic performance. Consequently, the true clinical utility of CHRPE as a screening tool in contemporary practice remains uncertain.

In the contemporary era of molecular diagnostics, the clinical role of CHRPE requires reappraisal. A systematic synthesis that accounts for methodological variability and genotype–phenotype correlations is necessary to clarify whether CHRPE retains meaningful diagnostic utility, particularly in risk stratification and resource-limited settings. The primary objective of this systematic review was to evaluate the diagnostic performance of CHRPE for identifying individuals with FAP or pathogenic APC mutations and to determine its potential clinical utility as an adjunctive phenotypic marker within contemporary screening strategies.

## Methods

This systematic review followed Preferred Reporting Items for Systematic Reviews and Meta-Analyses statement (PRISMA) guideline [20]. The study protocol was registered in the International Prospective Register of Systematic Reviews (PROSPERO; registration number CRD420251266287). The predefined review question was whether the presence of CHRPE can reliably identify individuals with FAP or pathogenic APC mutations and therefore contribute to risk stratification and screening.

### Search strategy and study eligibility

A systematic search was conducted in PubMed/MEDLINE, Embase, and Web of Science on August 30, 2025, to identify studies evaluating the association between CHRPE and FAP. No restrictions were applied regarding publication date. Only studies published in English and involving human subjects were considered. Search strategies combined MeSH, Emtree, and text words for FAP and CHRPE. Complete search strings for each database are provided in Table S1-S3.

Two reviewers (EAK and RD) independently screened all titles, abstracts, and full-text articles. Discrepancies were resolved through discussion until consensus was reached. Studies were eligible if they reported original clinical data, included individuals with confirmed or suspected FAP, or first-degree relatives at risk, and documented the presence or absence of CHRPE based on an ophthalmic examination. Exclusion criteria included case reports with fewer than five cases, review articles, conference abstracts without full data, editorials, non-English studies, and studies lacking ophthalmic evaluation.

### Data extraction and quality assessment

A standardized data extraction form was used by two reviewers (MS and RD) to collect study characteristics, patient demographics, CHRPE definitions, criteria for CHRPE positivity, methods of ophthalmic assessment, and outcome data. Conceptual and methodological heterogeneity across studies was assessed prior to any attempt at quantitative synthesis.

The methodological quality of included studies was assessed independently by two reviewers using the Quality Assessment of Diagnostic Accuracy Studies-2 (QUADAS-2) tool. The tool evaluates risk of bias and applicability concerns across four domains: patient selection, index test, reference standard, and flow and timing [21, 22]. 

### Qualitative synthesis

The target condition was defined as carriage of a pathogenic APC mutation. The reference standard was restricted to molecular genetic testing for APC mutations. For patient selection, studies were evaluated for consecutive or random recruitment, avoidance of case–control designs, and absence of inappropriate exclusions. Case–control studies and studies enrolling only APC mutation-positive individuals were judged to be at high risk of bias due to spectrum effects and limited applicability to screening populations. For the index test domain, studies were assessed for blinding of CHRPE assessment to genetic test results, pre-specification of CHRPE diagnostic criteria, and adequacy of ophthalmic examination methods. Differences in lesion definitions were not considered a source of bias unless diagnostic thresholds were modified after data collection. For the reference standard domain, only studies employing APC genetic testing were considered low risk. Studies relying solely on clinical diagnosis or family history were judged to be at high risk of bias. For flow and timing, studies were assessed for complete verification with both index and reference tests, appropriate timing between assessments, and inclusion of all enrolled participants in the final analysis. No study was rated as having a low risk of bias across all four assessed domains (Table S4).

Given that many included studies were conducted in tertiary referral centers or established FAP registries, spectrum bias may have influenced reported diagnostic performance. Case–control designs and enrichment with genetically confirmed mutation carriers are likely to overestimate specificity relative to unselected screening populations. Additionally, variability in blinding procedures and pre-specification of CHRPE diagnostic thresholds introduces potential observer and threshold bias. Substantial heterogeneity was also identified across multiple methodological domains, including CHRPE diagnostic definitions (ranging from presence of any lesion to structured criteria based on lesion morphology, number, size thresholds, bilaterality requirements, and composite scoring systems), ophthalmic examination techniques (including indirect ophthalmoscopy, slit-lamp biomicroscopy, fundus photography, optical coherence tomography, and multimodal imaging approaches), study populations (comprising confirmed FAP cases, APC mutation carriers, at-risk relatives, registry-based cohorts, and clinically diagnosed patients with variable reliance on colonoscopy and histopathology), reference standards (including clinical diagnosis, linkage analysis, early molecular testing approaches, APC sequencing, and contemporary next-generation sequencing methods), and study designs (including case–control, cohort, cross-sectional, family-based, and registry-based studies). Because these differences directly affect sensitivity and specificity estimates and violate key assumptions required for meaningful pooling of diagnostic accuracy measures, quantitative meta-analysis was deemed inappropriate and the findings are therefore presented as a structured qualitative synthesis.

## Results

**Fig. 1:**
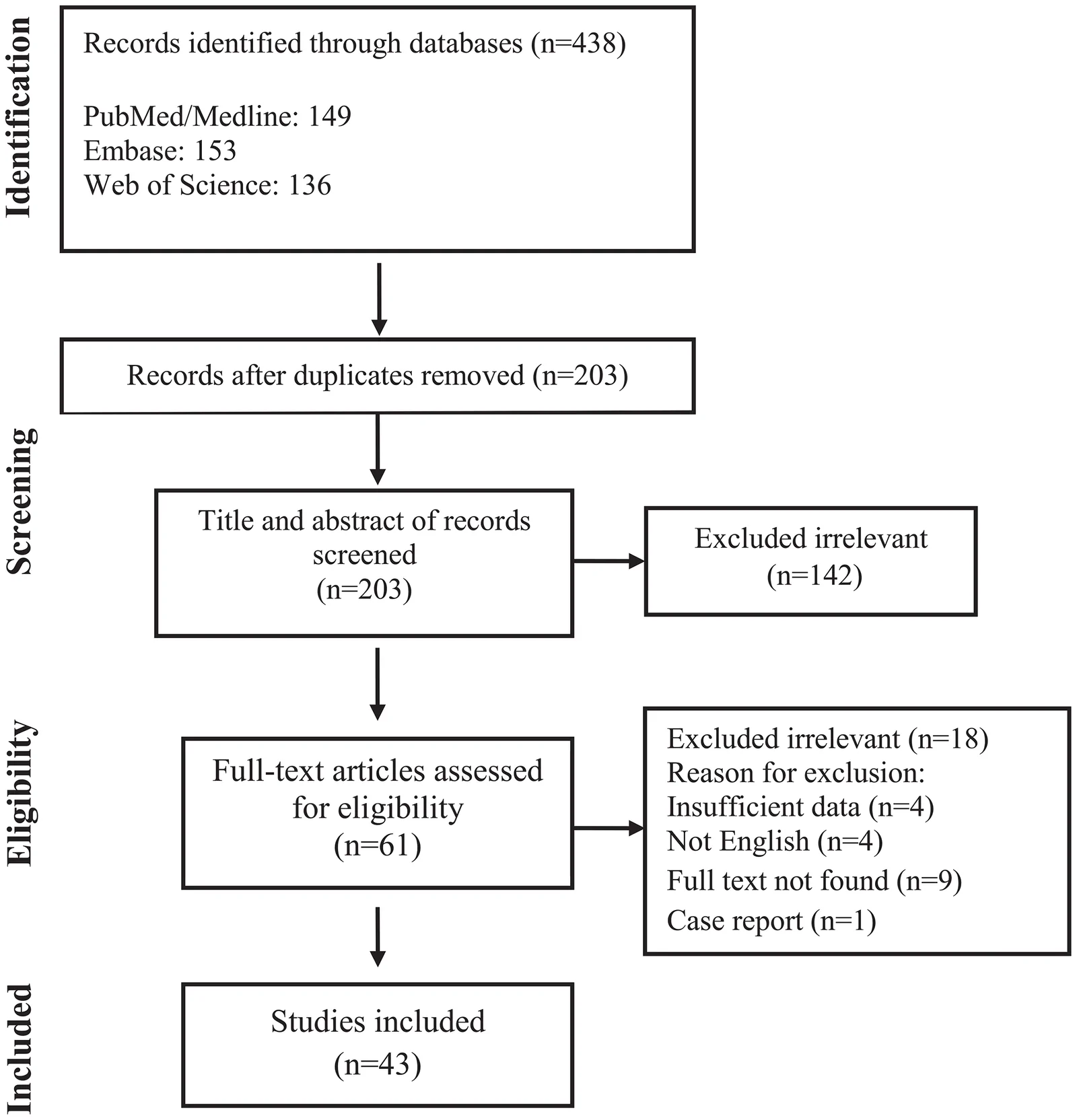
PRISMA flow diagram depicting the study identification, screening, and inclusion process for this systematic review

A total of 438 records were identified through the database searches. After removing duplicates, 203 unique records remained for title and abstract screening. Of these, 142 were excluded as irrelevant. The remaining 61 full-text articles were assessed for eligibility. Eighteen full-text articles were excluded for the following reasons: insufficient data (*n* = 4), not in English (*n* = 4), full text not found (*n* = 9), or case report (*n* = 1). Ultimately, 43 studies met the inclusion criteria and were included in the systematic review (Fig. 1).

These 43 studies included various designs, including case-control studies, retrospective and prospective cohort studies, cross-sectional studies, and case series. The main characteristics and key findings of the included studies are presented in Supplementary Tables 5 and 6.

The included studies represent more than thirty years of research across both ophthalmology and gastroenterology. Early publications often relied on descriptive clinical examination of the ocular fundus, while contemporary studies more frequently incorporated genetic testing and clearer phenotypic definitions. Across the body of literature, differences were evident in study design, sample size, participant characteristics, and the specific diagnostic criteria used to define CHRPE.

Although all studies focused on the association between CHRPE and FAP, there was substantial inconsistency in how lesions were described, measured, or classified. Some investigators defined CHRPE using broad descriptive terms such as flat pigmented lesions with sharp borders, while others applied structured morphologic classifications, including the Berk or Touriño systems [16, 17, 23–25]. The Touriño classification system categorizes CHRPE lesions into six morphological subtypes: (A) a well-demarcated oval pigmented lesion, commonly associated with a depigmented halo or a linear depigmented streak; (B) a lesion similar to type A but lacking a halo or linear depigmented streak; (C) a linear pigmented lesion with focal dot-like depigmentation; (D) a cylindrical pigmented lesion with peripheral depigmentation; (E) a small, dot-like pigmented lesion; and (F) an irregularly shaped pigmented lesion [25]. The morphological classification system adapted from Berk categorizes CHRPE lesions into four distinct subtypes. Type 1 consists of an oval pigmented lesion with a surrounding halo, Type 2 includes round, small pigmented dots, Type 3 refers to larger pigmented spots compared with Type 2, and Type 4 comprises large, atrophic pigmented lesions. In this classification, Types 1 and 2 are considered high-risk indicators for FAP [26]. 

Studies applying the Touriño classification have reported that specific subtypes, particularly types A and B, show a strong association with FAP. These studies consistently demonstrated sensitivities ranging from 80% to 90% and a specificity of 100% when compared with healthy controls and individuals with sporadic colorectal cancer in certain populations [16]. 

Several studies proposed quantitative diagnostic thresholds based on disc-diameter measurements or lesion counts, contributing to substantial variability in reported diagnostic performance. Lesion count was a key component in many definitions of CHRPE positivity. While some studies considered the presence of two or more lesions as abnormal, others required at least four lesions to classify an individual as CHRPE-positive in the context of FAP screening [3, 6, 9, 27–31]. Bilaterality was frequently incorporated as an additional criterion, as it increased the likelihood of identifying APC mutation carriers [6, 31]. A smaller number of studies applied composite scoring systems that integrated lesion size and number into a single metric [30, 32]. 

With respect to lesion pigmentation, most studies reported that approximately 80–100% of CHRPE lesions observed in patients with FAP were hyperpigmented [18, 31, 33–36]. 

Due to differences in diagnostic criteria and assessment protocols, the reported sensitivity of CHRPE varied considerably across studies. In patients with established FAP, sensitivity ranged from 40% to 100%. Among individuals at risk for developing FAP, sensitivity ranged from 57% to 100%, while specificity varied between 58% and 100%, depending on the diagnostic definition applied. Studies employing more stringent criteria, particularly those requiring a minimum lesion count or bilateral involvement, generally reported higher specificity at the expense of reduced sensitivity [15, 29, 30, 35, 37]. 

Despite the methodological diversity, the overall findings of the included studies were highly consistent. CHRPE demonstrated very high specificity for FAP or APC mutation carriage across almost all data sets. For example, Olea et al. reported that broad definitions of CHRPE produced sensitivity values close to 90% but resulted in lower specificity [15]. When definitions were restricted to large lesions or bilateral involvement, specificity rose to 97% or higher, while sensitivity decreased in a predictable manner [15, 23]. 

Similar trends were seen in studies that focused on lesion number. Definitions that required at least four lesions consistently produced specificity values of 100%. These definitions also produced perfect positive predictive values, reflecting the rarity of multiple CHRPE lesions in the general population. Sensitivity values under these stricter criteria typically ranged from approximately 66 to nearly 90%. Burn et al. provided further confirmation of this pattern [29]. When CHRPE positivity was defined as the presence of four or more lesions, sensitivity was nearly 88% and both specificity and positive predictive value were 100% [6, 15, 35]. Increasing the threshold to six lesions reduced sensitivity modestly but retained perfect specificity [29]. 

When studies were stratified by reference standard, studies incorporating APC mutation testing, sequencing, or molecular risk assessment, CHRPE generally retained high specificity. Where reported, specificity in these studies was consistently high, ranging from approximately 96% to 100%, while sensitivity remained variable, ranging from about 64% to 90%. This variability is biologically plausible because CHRPE expression is influenced by APC mutation location and ocular penetrance. In contrast, studies based primarily on clinical diagnosis, colonoscopy, registry ascertainment, or family history showed a wider range of diagnostic performance, with sensitivity ranging from as low as 14.2% in single-kindred studies to approximately 89% in larger registry-based cohorts, while specificity ranged from about 61% to 100% depending on how strictly CHRPE was defined and the characteristics required for a positive finding. In both groups, the absence of CHRPE was insufficient to exclude FAP or APC mutation carriage.

## Discussion

This systematic review demonstrates that CHRPE, when defined using stringent morphologic and quantitative criteria, exhibits consistently high specificity for APC mutation carriage. Across more than three decades of research, the presence of multiple, bilateral, or large lesions was strongly associated with FAP, whereas broader or less structured definitions resulted in reduced diagnostic precision [23, 38]. 

The observed variability in sensitivity reflects both methodological heterogeneity and biological diversity in phenotypic expression. Not all APC mutation carriers develop CHRPE, and lesion expression appears influenced by mutation location. Mutations located between codons 446 and 1328 have been most consistently associated with retinal lesions, whereas variants outside this region may result in classic intestinal manifestations without ocular findings [1, 5, 39, 40]. This genotype–phenotype correlation provides a biological explanation for incomplete sensitivity and underscores why a normal fundus examination cannot exclude FAP.

Interpretation of earlier studies must also consider historical limitations in genetic confirmation. Before routine APC testing, mutation status was frequently inferred from clinical criteria or family history, introducing potential misclassification. Such limitations likely contributed to variability in both sensitivity and specificity across early publications. Furthermore, studies incorporating molecular confirmation demonstrate more consistent diagnostic patterns and clearer correlations between lesion morphology and mutation carriage [5, 40, 41]. 

These findings are consistent with the only recent systematic review on CHRPE in FAP. Bonnet et al. reported high specificity and variable sensitivity of CHRPE, emphasizing the influence of lesion definition and study population, and confirming that multiple or bilateral lesions are most strongly associated with APC mutation carriage. They also highlighted that absence of CHRPE does not exclude disease [26]. Notably, there are limited contemporary systematic reviews focused specifically on CHRPE, underscoring the need for updated stratified analyses using standardized criteria and molecularly confirmed cohorts.

From a clinical perspective, CHRPE should not be viewed as a replacement for molecular testing but rather as a highly specific adjunctive marker. Its value lies primarily in risk stratification. In individuals from affected families, the identification of multiple or bilateral lesions substantially increases the likelihood of mutation carriage and may justify prioritization of genetic testing and gastrointestinal surveillance. Conversely, absence of CHRPE does not reliably exclude disease.

The potential utility of CHRPE is particularly relevant in selected contexts. In pediatric patients, where endoscopic screening may carry greater procedural burden, noninvasive ophthalmic evaluation offers a low-risk adjunctive assessment. Similarly, in resource-limited settings where access to comprehensive genetic testing is restricted, fundus examination may provide an accessible phenotypic clue prompting further evaluation [10, 12, 19]. 

Importantly, ophthalmologists may occasionally encounter individuals without a known family history of FAP who present with characteristic CHRPE lesions. Recognition of high-risk lesion patterns in such patients may facilitate earlier systemic investigation and reduce diagnostic delay. However, isolated or solitary pigmented fundus lesions are relatively common in the general population and should not trigger unnecessary systemic evaluation in the absence of characteristic multiplicity or bilaterality [36, 42]. 

An important limitation of the available evidence is spectrum bias. Many studies were conducted in tertiary referral centers, hereditary cancer registries, or genetically enriched family cohorts. Such populations differ substantially from unselected screening populations because disease prevalence is markedly higher and phenotypic manifestations are often more pronounced. Consequently, diagnostic performance estimates, particularly specificity and positive predictive value, may be overestimated relative to general clinical practice. This limitation should be considered when interpreting the apparent diagnostic accuracy of CHRPE.

Although heterogeneity in lesion definitions limited quantitative meta-analysis, qualitative findings were consistent in demonstrating high specificity under stringent criteria. Future research would benefit from standardized morphologic definitions, incorporation of disc-diameter–based measurement thresholds, multimodal imaging documentation, and systematic genotype correlation. Prospective studies in genetically characterized cohorts are needed to define the incremental diagnostic value of CHRPE within contemporary screening algorithms.

## Conclusion

CHRPE demonstrates consistently high specificity for APC mutation carriage when stringent morphologic and quantitative criteria are applied. However, incomplete sensitivity and substantial historical heterogeneity limit its role as a standalone diagnostic tool. It should therefore be regarded as an adjunctive phenotypic marker rather than a diagnostic test. In the era of molecular diagnostics, CHRPE functions best as a supportive feature for risk stratification, particularly in pediatric populations and resource-constrained settings. Standardized lesion definitions and prospective genotype-driven studies are required to clarify its precise role within modern FAP screening strategies.

## Supplementary Information

Below is the link to the electronic supplementary material.

Supplementary Material 1

## Data Availability

All data supporting the findings of this study are available within the paper and its Supplementary Information.

## Abbreviations

APC: Adenomatous Polyposis Coli
CHRPE: Congenital Hypertrophy of the Retinal Pigment Epithelium
DNA: Deoxyribonucleic Acid
FAP: Familial Adenomatous Polyposis
MeSH: Medical Subject Headings
PRISMA: Preferred Reporting Items for Systematic Reviews and Meta-Analyses
PROSPERO: International Prospective Register of Systematic Reviews
QUADAS-2: Quality Assessment of Diagnostic Accuracy Studies-2
