# Supplementary material for: Diagnostic value of congenital hypertrophy of the retinal pigment epithelium in familial adenomatous polyposis: a systematic review
Source: Int J Retina Vitreous. 2026 Jun 23;12:117. doi: 10.1186/s40942-026-00886-3 (PMC13548638; doi:10.1186/s40942-026-00886-3)
Supplement: Supplementary file 1 [file 40942_2026_886_MOESM1_ESM.docx]

Supplementary Tables:

Table S1. Search strategy used for PubMed/MEDLINE.

| #1 | (((((Adenomatous Polyposis Coli[MeSH Terms]) OR ("Familial adenomatous polyposis"[Title/Abstract])) OR (FAP[Title/Abstract])) OR ("Adenomatous Intestinal Polyposis"[Title/Abstract])) OR ("Familial Intestinal Polyposis"[Title/Abstract])) OR ("Familial Multiple Polyposis"[Title/Abstract]) (13878) |
| --- | --- |
| #2 | ("Congenital Hypertrophy of the Retinal Pigment Epithelium"[Title/Abstract]) OR (CHRPE[Title/Abstract]) (257) |
| #3 | #1 AND #2 (149) |

Table S2. Search strategy used for Embase

| #1 | 'familial adenomatous polyposis'/mj OR fap:ti,ab,kw OR 'adenomatous polyposis coli':ti,ab,kw OR 'adenomatous intestinal polyposis':ti,ab,kw OR 'familial intestinal polyposis':ti,ab,kw OR 'familial multiple polyposis':ti,ab,kw (17598) |
| --- | --- |
| #2 | 'congenital hypertrophy of the retinal pigment epithelium':ti,ab,kw OR chrpe:ti,ab,kw (318) |
| #3 | #1 AND #2 (153) |

Table S3. Search strategy used for Web of Science

| #1 | "Familial adenomatous polyposis" (Topic) or FAP (Topic) or "Adenomatous Polyposis Coli" (Topic) or "Adenomatous Intestinal Polyposis" (Topic) or "Familial Intestinal Polyposis" (Topic) or "Familial Multiple Polyposis" (Topic) (18352) |
| --- | --- |
| #2 | "Congenital Hypertrophy of the Retinal Pigment Epithelium" (Topic) or CHRPE (Topic) (222) |
| #3 | #1 AND #2 (136) |

Table S4. Results of the risk of bias and applicability concerns assessment across the included studies.

| **First author (Year)** | **Risk of Bias** | | | | **Applicability Concerns** | | |
| --- | --- | --- | --- | --- | --- | --- | --- |
|  | **Patient Selection** | **Index Test** | **Reference Standard** | **Flow and Timing** | **Patient Selection** | **Index Test** | **Reference Standard** |
| Berk et al. (1988)(1) | ☹ | 😊 | ☹ | ☹ | ☹ | 😊 | ☹ |
| Traboulsi et al. (1988)(2) | ☹ | 😊 | ☹ | ☹ | 😊 | 😊 | ☹ |
| Garber et al. (1988)(3) | ☹ | ? | ☹ | ☹ | ☹ | ? | ☹ |
| Romania et al. (1989)(4) | ☹ | ? | ☹ | ☹ | 😊 | 😊 | ☹ |
| Chapman et al. (1989)(5) | ☹ | ☹ | ☹ | ☹ | ☹ | 😊 | ☹ |
| Iwama et al. (1990)(6) | ☹ | ? | ☹ | ☹ | ? | 😊 | ☹ |
| Traboulsi et al. (1990)(7) | ☹ | 😊 | ☹ | ☹ | ☹ | 😊 | ☹ |
| Heyen et al. (1990)(8) | ☹ | ? | ☹ | ☹ | 😊 | ☹ | ☹ |
| Burn et al. (1991)(9) | ☹ | ☹ | ☹ | ☹ | ☹ | ☹ | ☹ |
| Giardiello et al. (1991)(10) | ☹ | 😊 | ☹ | ☹ | ☹ | 😊 | ☹ |
| Houlston et al. (1992)(11) | ☹ | ? | ☹ | ☹ | ☹ | 😊 | ☹ |
| Moore et al. (1992)(12) | ☹ | 😊 | ☹ | ☹ | 😊 | 😊 | ☹ |
| Shields et al. (1992)(13) | ☹ | 😊 | ☹ | ☹ | ☹ | 😊 | ☹ |
| Morton et al. (1992)(14) | ☹ | 😊 | ☹ | ☹ | 😊 | 😊 | ☹ |
| Hickey-Dwyer et al. (1993)(15) | ☹ | ? | ☹ | ☹ | ? | 😊 | ☹ |
| Bertario et al. (1993)(16) | ☹ | 😊 | ☹ | ☹ | ☹ | 😊 | ☹ |
| Maher et al. (1993)(17) | 😊 | ? | ? | ☹ | 😊 | 😊 | 😊 |
| Hunt et al. (1994)(18) | ☹ | 😊 | ☹ | ☹ | ☹ | 😊 | ☹ |
| Santos et al. (1994)(19) | ☹ | ? | ☹ | ☹ | ☹ | 😊 | ☹ |
| Hodgson et al. (1994)(20) | ☹ | 😊 | ☹ | ☹ | ☹ | 😊 | ☹ |
| Campbell et al. (1994)(21) | ☹ | ☹ | ☹ | ☹ | ☹ | 😊 | ☹ |
| De Pietri et al. (1995)(22) | ☹ | ? | ☹ | ? | ☹ | 😊 | 😊 |
| Grobbelaar et al. (1996)(23) | ? | ? | 😊 | ☹ | ? | 😊 | 😊 |
| Rossato et al. (1996)(24) | ☹ | 😊 | ☹ | ☹ | 😊 | 😊 | ☹ |
| Valanzano et al. (1996)(25) | ? | ? | ? | ☹ | 😊 | 😊 | 😊 |
| Olea et al. (1996)(26) | ☹ | ? | ☹ | ☹ | ☹ | 😊 | ☹ |
| Traboulsi et al. (1996)(27) | ☹ | ? | 😊 | ? | ☹ | 😊 | 😊 |
| Tiret et al. (1997)(28) | ☹ | ☹ | ☹ | ☹ | 😊 | 😊 | 😊 |
| Reck et al. (1997)(29) | ☹ | ☹ | ? | ? | ☹ | 😊 | 😊 |
| Ruhswurm et al. (1998)(30) | ☹ | 😊 | ? | ☹ | 😊 | 😊 | ☹ |
| Gebert et al. (1999)(31) | ? | 😊 | ? | ☹ | 😊 | 😊 | 😊 |
| Cao et al. (2000)(32) | ? | ? | ? | ☹ | 😊 | ? | 😊 |
| Pang et al. (2000)(33) | ☹ | ? | ? | ? | ☹ | 😊 | 😊 |
| Cetta et al. (2000)(34) | ☹ | ? | ? | ☹ | ☹ | 😊 | 😊 |
| Chen et al. (2006)(35) | ☹ | ? | ? | ☹ | ☹ | 😊 | 😊 |
| Cai SR et al. (2007)(36) | ☹ | ? | ☹ | ☹ | ☹ | ? | ☹ |
| Garzón-Benavides et al. (2010)(37) | ? | ? | ? | ☹ | ☹ | 😊 | 😊 |
| Burger et al. (2011)(38) | ? | ? | ? | ☹ | ☹ | 😊 | 😊 |
| Nusliha et al. (2014)(39) | ☹ | ? | ☹ | ☹ | ☹ | 😊 | ☹ |
| Mirinezhad et al. (2018)(40) | ☹ | ☹ | ☹ | ☹ | ☹ | 😊 | ☹ |
| de Oliveira et al. (2019)(41) | ? | ☹ | ? | ? | ☹ | 😊 | 😊 |
| Cai et al. (2022)(42) | ☹ | 😊 | ? | ☹ | 😊 | 😊 | 😊 |
| Carvalho et al. (2023)(43) | ☹ | ? | ☹ | ☹ | 😊 | 😊 | ☹ |

Table S5. Main characteristics of the 43 studies included in the systematic review.

| First author (Year) | Study design | CHRPE positivity criteria | Ophthalmic assessment | FAP assessment |
| --- | --- | --- | --- | --- |
| Berk et al. (1988) | Cross-sectional study | Four lesion types; multiple bilateral lesions characteristic | Indirect ophthalmoscopy | Colonoscopy; ECM assessment |
| Traboulsi et al. (1988) | Cross-sectional ophthalmic survey | ≥3 CHRPE patches | Fundus examination + photography | Clinical polyposis diagnosis |
| Garber et al. (1988) | Registry-based survey | Presence of CHRPE lesions | Ophthalmologic examination | Colonoscopy; family history |
| Romania et al. (1989) | Cross-sectional ophthalmic survey | Bilateral ≥4 lesions | Wide-angle fundus photography | Colonoscopy; ECM assessment |
| Chapman et al. (1989) | Cross-sectional study | Multiple CHRPE lesions; controls ≤2 lesions | Indirect ophthalmoscopy | Colonoscopy, pedigree analysis |
| Iwama et al. (1990) | Cross-sectional family-based study | Pigmented retinal lesions classified into three types | Ophthalmoscopy with retinal mapping | Clinical FAP diagnosis; upper GI evaluation |
| Traboulsi et al. (1990) | Prospective observational study | ≥4 lesions in one/both eyes | Indirect ophthalmoscopy | Colonoscopy; ECM evaluation |
| Heyen et al. (1990) | Cross-sectional ophthalmic survey | ≥4 bilateral lesions | Fundus photography + drawings | Colonoscopy + ECM assessment |
| Burn et al. (1991) | Cross-sectional diagnostic screening study | >3 lesions diagnostic; ≥6 essentially diagnostic | Indirect ophthalmoscopy | Registry, bowel screening, linkage analysis |
| Giardiello et al. (1991) | Cross-sectional diagnostic study | ≥1 lesion in both retinas | Indirect ophthalmoscopy + photography | Endoscopy/surgery; registry ascertainment |
| Houlston et al. (1992) | Cross-sectional study | Multiple lesions (≥4) considered significant | Indirect ophthalmoscopy | Colonoscopy, pedigree analysis |
| Moore et al. (1992) | Population-based ophthalmic survey | ≥1 lesion; subgroup >3 lesions | Indirect ophthalmoscopy | Clinical/DNA risk assessment |
| Shields et al. (1992) | Retrospective observational study | Solitary/grouped CHRPE lesions | Fundus examination + photography | Family history verification |
| Morton et al. (1992) | Prospective population-based screening study | ≥2 of: bilaterality, ≥3 lesions, lesion >0.3 DD | Indirect ophthalmoscopy and slit lamp examination | Colonoscopy and linkage analysis |
| Hickey-Dwyer et al. (1993) | Cross-sectional family-based study | Four lesion morphologies classified | Indirect ophthalmoscopy + 90D lens | Colonoscopy, pedigree analysis |
| Bertario et al. (1993) | Cross-sectional study | Multiple lesions or single lesion >1 DD | Indirect/direct ophthalmoscopy; fundus photography | Colonoscopy; ECM assessment |
| Maher et al. (1993) | Population-based genetic study | >3 CHRPE lesions | Indirect ophthalmoscopy | Endoscopy + linkage analysis |
| Hunt et al. (1994) | Comparative cross-sectional study | Any lesion positive; multiple ≥3 lesions | Indirect ophthalmoscopy | Familial CRC assessment |
| Santos et al. (1994) | Cross-sectional study | Multiple bilateral lesions | Ophthalmic exam + ERG/EOG/angiography | Clinical FAP diagnosis |
| Hodgson et al. (1994) | Cross-sectional study | Type A; oval pigmented lesion with depigmented halo. Additional types (B-E) recorded. | indirect ophthalmoscopy | Colonoscopy + linkage/mutation testing |
| Campbell et al. (1994) | Cross-sectional diagnostic study | Multiple grouped lesions or solitary large lesion | Indirect ophthalmoscopy + retinal mapping | Registry ascertainment; clinical FAP diagnosis |
| De Pietri et al. (1995) | Cross-sectional registry-based study | Presence of unilateral/bilateral CHRPE lesions | Dilated fundus examination | Endoscopy, surgery/pathology, APC testing |
| Grobbelaar et al. (1996) | Genotype–phenotype correlation study | ≥1 lesion; categorized as 1–4 vs >4 lesions; large lesions >0.5 DD | Indirect ophthalmoscopy | APC exon 10 PCR, SSCP, sequencing, segregation analysis |
| Rossato et al. (1996) | Cross-sectional study | ≥1 characteristic lesion; lesion size classified by DD | Fundus exam + photography | Colonoscopy; extracolonic assessment |
| Valanzano et al. (1996) | Cross-sectional study | Score ≥3 based on lesion morphology/size | Dilated fundus exam + photography | Colonoscopy + APC testing |
| Olea et al. (1996) | Cross-sectional observational study | Large lesions and >4 lesions most predictive | Goldmann lens retinal examination | Rectosigmoidoscopy |
| Traboulsi et al. (1996) | Genotype–phenotype correlation study | ≥3 lesions/patient or characteristic configuration | Dilated fundus examination | APC mutation analysis |
| Tiret et al. (1997) | Cross-sectional observational study | ≥4 lesions or ≥2 including one large lesion | Dilated fundus exam + photography | Colonoscopy + APC analysis |
| Reck et al. (1997) | Genotype–phenotype correlation study | CHRPE coefficient >3 | Indirect ophthalmoscopy | APC mutation analysis |
| Ruhswurm et al. (1998) | Prospective ophthalmic-genetic study | ≥4 small or ≥1 large (>0.5 DD) lesion | Slit lamp + ophthalmoscopy | Colonoscopy + mutation analysis |
| Gebert et al. (1999) | Combined molecular-clinical study | ≥1 large lesion or ≥4 bilateral lesions | Fundus examination | PTT, sequencing, clinical screening |
| Cao et al. (2000) | Genotype–phenotype correlation study | Presence/absence of CHRPE | Ophthalmic examination | Colonoscopy, pedigree analysis, PTT, sequencing |
| Pang et al. (2000) | Family-based genotype study | Type A; oval pigmented lesion with depigmented halo. Additional types (B-E) recorded. | Binocular indirect ophthalmoscopy | Colonoscopy + APC sequencing |
| Cetta et al. (2000) | Multicenter genotype study | Typical CHRPE lesions with halo | Fundus examination | APC sequencing + thyroid pathology |
| Chen et al. (2006) | Prospective cohort study | Multiple bilateral lesions with depigmented halo | Indirect ophthalmoscopy + slit lamp | APC mutation analysis; MSI testing |
| Cai SR et al. (2007) | Cross-sectional family-based study | Presence of CHRPE | Ophthalmologic examination | Clinical classification, pedigree analysis |
| Garzón-Benavides et al. (2010) | Cross-sectional registry-based case series | Multiple bilateral lesions | Ophthalmoscopy | Clinical/molecular FAP diagnosis; APC testing |
| Burger et al. (2011) | Prospective dermatologic-genetic study | Berk classification (types A–D) | Indirect ophthalmoscopy | APC mutation testing |
| Nusliha et al. (2014) | Cross-sectional registry-based study | Single/multiple unilateral or bilateral lesions | Slit lamp + indirect ophthalmoscopy | Colonoscopy/sigmoidoscopy |
| Mirinezhad et al. (2018) | Cross-sectional study | Lesions documented by number, size, laterality, form, and location. No strict numerical cutoff. | Slit lamp, indirect ophthalmoscopy, OCT | Colonoscopy + pathology |
| de Oliveira et al. (2019) | Prospective single-center study | No detailed lesion criteria specified | Ophthalmoscopy | Colonoscopy + APC sequencing |
| Cai et al. (2022) | Cross-sectional study | Six categories (Tourino’s classification) | Indirect ophthalmoscopy + fundus photography and fluorescence angiography | Colonoscopy records + pathology diagnosis or family history +  APC sequencing |
| Carvalho et al. (2023) | Cross-sectional family-based case series | Bilateral multiple lesions with fish-tail depigmentation | Slit lamp + indirect ophthalmoscopy | Colonoscopy + APC NGS sequencing |
| CHRPE: Congenital Hypertrophy of the Retinal Pigment Epithelium, FAP: Familial Adenomatous Polyposis, APC: Adenomatous polyposis coli, ECM: Extracolonic Manifestation, HNPCC: Hereditary Nonpolyposis Colorectal Cancer, DD: Disc Diameter, CRC: Colorectal Cancer, ERG: Electroretinography, EOG: Electrooculography, PCR: Polymerase Chain Reaction, PTT: Protein Truncation Test, PTC: Papillary Thyroid Cancer, MSI: Microsatellite Instability, OCT: Optical Coherence Tomography, NGS: Next-Generation Sequencing. | | | | |

Table S6. Key findings and performance metrics of the 43 studies included in the systematic review.

| First author (Year) | Sample size (patients) | Key findings | Performance Metrics |
| --- | --- | --- | --- |
| Berk et al. (1988) | n=40 FAP + 11 at-risk + 8 controls | Useful early carrier marker | Sensitivity 87.5%; specificity NR; PPV NR; NPV NR. |
| Traboulsi et al. (1988) | n=174 from 33 families | CHRPE specific for Gardner syndrome phenotype | Sensitivity NR; specificity NR; PPV NR; NPV NR. |
| Garber et al. (1988) | n=25 hepatoblastoma cases | CHRPE common in hepatoblastoma survivors from FAP families | Sensitivity NR; specificity NR; PPV NR; NPV NR. |
| Romania et al. (1989) | n=97 FAP + 56 at-risk | CHRPE predictive in 65% of kindreds | Sensitivity NR; specificity NR; PPV NR; NPV NR. |
| Chapman et al. (1989) | n=40 FAP + 35 controls | 100% of carriers had multiple lesions | Sensitivity NR; specificity NR; PPV NR; NPV NR. |
| Iwama et al. (1990) | n=43 FAP + 6 relatives | CHRPE in 58%; more common in Gardner syndrome | Sensitivity NR; specificity NR; PPV NR; NPV NR. |
| Traboulsi et al. (1990) | n=34 at-risk | Strong predictive value for future polyposis | Sensitivity NR; specificity NR; PPV NR; NPV NR. |
| Heyen et al. (1990) | n=97 FAP + 51 at-risk | Earlier polyp onset in CHRPE-positive kindreds | Sensitivity NR; specificity NR; PPV NR; NPV NR. |
| Burn et al. (1991) | n=66 carriers + 151 at-risk + 94 controls | Combined CHRPE/DNA improved carrier prediction | Sensitivity NR; specificity NR; PPV NR; NPV NR. |
| Giardiello et al. (1991) | n=43 FAP + 12 unaffected relatives | Combined ocular/jaw markers improved diagnostic accuracy | Combined ocular+jaw: sensitivity 77%; specificity 92%; PPV 97%; NPV 52%. CHRPE-only: sensitivity 58%; specificity 100%; PPV 100%; NPV 40%. |
| Houlston et al. (1992) | n=21 | Multiple CHRPE in 14%; possible APC involvement in non-classic syndromes | Sensitivity NR; specificity NR; PPV NR; NPV NR. |
| Moore et al. (1992) | n=33 FAP + 33 at-risk | Strong interfamilial consistency | Sensitivity NR; specificity NR; PPV NR; NPV NR. |
| Shields et al. (1992) | n=132 | No association with FAP/Gardner syndrome | Sensitivity NR; specificity NR; PPV NR; NPV NR. |
| Morton et al. (1992) | n=32 FAP + 43 at-risk + 10 HNPCC | CHRPE is a useful predictive marker for FAP, but should be used with DNA analysis and bowel examination. | Sensitivity 84%; specificity >=94%; PPV NR; NPV NR. |
| Hickey-Dwyer et al. (1993) | n=14 FAP + 14 at-risk | CHRPE unreliable as standalone marker | Sensitivity 14.2%; specificity NR; PPV NR; NPV NR. |
| Bertario et al. (1993) | n=48 FAP + 30 at-risk | CHRPE is a common ECM | Sensitivity 70%; specificity 90%; PPV 92%; NPV NR. |
| Maher et al. (1993) | n=54 affected + 48 at-risk | Marked interfamilial variability | Sensitivity NR; specificity NR; PPV NR; NPV NR. |
| Hunt et al. (1994) | n=33 familial + 36 sporadic + 32 controls | Multiple CHRPE associated with familial CRC | Sensitivity NR; specificity NR; PPV NR; NPV NR. |
| Santos et al. (1994) | n=7 | Functional retinal testing largely normal | Sensitivity NR; specificity NR; PPV NR; NPV NR. |
| Hodgson et al. (1994) | n=118 FAP + 80 low-risk patients | Type A lesions highly predictive | Sensitivity NR; specificity NR; PPV NR; NPV NR. |
| Campbell et al. (1994) | n=43 FAP + 27 at-risk + 40 controls | Morphology more important than count | Sensitivity 79%; specificity 95%; PPV NR; NPV NR. |
| De Pietri et al. (1995) | n=48 FAP vs 200 controls; bilateral exam | No clear genotype correlation | Sensitivity 64%; specificity 99%; PPV 97%; NPV NR. |
| Grobbelaar et al. (1996) | n=17 FAP + 15 relatives; ophthalmic exam in 9 | Novel APC codon 441 mutation; all mutation-positive examined subjects had CHRPE | Sensitivity NR; specificity NR; PPV NR; NPV NR. |
| Rossato et al. (1996) | n=36 relatives + 160 controls | Large lesions highly specific for FAP | Sensitivity NR; specificity NR; PPV NR; NPV NR. |
| Valanzano et al. (1996) | n=39 FAP + 53 at-risk; bilateral exam | novel CHRPE score based on morphologic and dimensional criteria. Exon 15 mutation carriers showed CHRPE more consistently than exon 9 carriers. | Sensitivity 72.88%; specificity 96.29%; PPV 97.72%; NPV 61.90%. |
| Olea et al. (1996) | n=29 FAP + 38 at-risk + 26 controls | Large lesions most discriminative | Large CHRPE lesions: sensitivity 82%; specificity 97%; PPV 96%; NPV 88%. Any CHRPE: sensitivity 89%; specificity 76%; PPV 74%; NPV 90%. |
| Traboulsi et al. (1996) | n=21 families | Ocular phenotype strongly mutation-dependent | Sensitivity NR; specificity NR; PPV NR; NPV NR. |
| Tiret et al. (1997) | n=144 from 85 families | Fundus examination can help detect FAP gene carriers early in families with positive ocular findings. | Sensitivity 67.7%; specificity 100%; PPV 100%; NPV NR. |
| Reck et al. (1997) | n=12 | Mutations before exon 9 associated with absent CHRPE | Sensitivity NR; specificity NR; PPV NR; NPV NR. |
| Ruhswurm et al. (1998) | n=20 FAP + 19 at-risk | Complete agreement with molecular risk estimation | Sensitivity NR; specificity NR; PPV NR; NPV NR. |
| Gebert et al. (1999) | n=205 patients from 85 families | Strong correlation with APC codons 463–1444 | Sensitivity NR; specificity NR; PPV NR; NPV NR. |
| Cao et al. (2000) | n=115 from 28 families; detailed phenotype in 72 | CHRPE consistently associated with APC codons 463–1387 | Sensitivity NR; specificity NR; PPV NR; NPV NR. |
| Pang et al. (2000) | Two families; bilateral exam | CHRPE present despite mutations before codon 413 | Sensitivity NR; specificity NR; PPV NR; NPV NR. |
| Cetta et al. (2000) | n=15 FAP-PTC patients | CHRPE strongly linked to codons 463–1387 | Sensitivity NR; specificity NR; PPV NR; NPV NR. |
| Chen et al. (2006) | n=9 FAP + 37 controls + 36 test cohort | CHRPE useful for FAP but not non-polyposis CRC | Sensitivity NR; specificity NR; PPV NR; NPV NR. |
| Cai SR et al. (2007) | n=27 families; CHRPE assessed in 16 families | CHRPE present in 93.8% of assessed families | Sensitivity NR; specificity NR; PPV NR; NPV NR. |
| Garzón-Benavides et al. (2010) | n=77 from 33 families; ophthalmic exam in 35 | CHRPE in 65.7%; strongly associated with APC codons 454–1019 | Sensitivity NR; specificity NR; PPV NR; NPV NR. |
| Burger et al. (2011) | n=56 FAP + 116 controls | CHRPE in 43.1%; weak genotype correlation | Sensitivity NR; specificity NR; PPV NR; NPV NR. |
| Nusliha et al. (2014) | n=17 FAP + 13 at-risk; bilateral exam | The study supports CHRPE as a low-cost screening method but in a small registry cohort. | Sensitivity 76%; specificity 92%; PPV 93%; NPV 75%. |
| Mirinezhad et al. (2018) | n=23 FAP + 26 relatives | Multiple CHRPE lesions are sensitive and specific clinical markers for FAP in the Iranian registry population. | Sensitivity 78.3%; specificity 61.5%; PPV 64.3%; NPV 76.2%. |
| de Oliveira et al. (2019) | n=99 from 35 families | CHRPE associated with APC exon 15 variants | Sensitivity NR; specificity NR; PPV NR; NPV NR. |
| Cai et al. (2022) | n= 97  from 23 families | CHRPE is a useful screening marker for FAP and APC mutation carriers, but while positive findings support further evaluation, negative results do not exclude disease. | Sensitivity 90.4%; specificity 100%; PPV NR; NPV NR. |
| Carvalho et al. (2023) | n=45 examined from 95-member pedigree | CHRPE strongly correlated with novel APC mutation | Sensitivity NR; specificity NR; PPV NR; NPV NR. |
| CHRPE: Congenital Hypertrophy of the Retinal Pigment Epithelium, FAP: Familial Adenomatous Polyposis, APC: Adenomatous polyposis coli, ECM: Extracolonic Manifestation, HNPCC: Hereditary Nonpolyposis Colorectal Cancer, CRC: Colorectal Cancer, PTC: Papillary Thyroid Cancer, NR: Not reported, PPV: Positive predictive value, NPV: Negative predictive value. | | | |

Supplementary References:

1.	Berk T, Cohen Z, McLeod RS, Parker JA. Congenital hypertrophy of the retinal pigment epithelium as a marker for familial adenomatous polyposis. Diseases of the colon and rectum. 1988;31(4):253-7.

2.	Traboulsi EI, Maumenee IH, Krush AJ, Giardiello FM, Levin LS, Hamilton SR. Pigmented ocular fundus lesions in the inherited gastrointestinal polyposis syndromes and in hereditary nonpolyposis colorectal cancer. Ophthalmology. 1988;95(7):964-9.

3.	Garber JE, Li FP, Kingston JE, Krush AJ, Strong LC, Finegold MJ, et al. Hepatoblastoma and familial adenomatous polyposis. Journal of the National Cancer Institute. 1988;80(20):1626-8.

4.	Romania A, Zakov ZN, McGannon E, Schroeder T, Heyen F, Jagelman DG. CONGENITAL HYPERTROPHY OF THE RETINAL-PIGMENT EPITHELIUM IN FAMILIAL ADENOMATOUS POLYPOSIS. Ophthalmology. 1989;96(6):879-84.

5.	Chapman PD, Church W, Burn J, Gunn A. Congenital hypertrophy of retinal pigment epithelium: a sign of familial adenomatous polyposis. BMJ (Clinical research ed). 1989;298(6670):353-4.

6.	Iwama T, Mishima Y, Okamoto N, Inoue J. Association of congenital hypertrophy of the retinal pigment epithelium with familial adenomatous polyposis. The British journal of surgery. 1990;77(3):273-6.

7.	Traboulsi EI, Murphy SF, Delacruz ZC, Maumenee IH, Green WR. A CLINICOPATHOLOGICAL STUDY OF THE EYES IN FAMILIAL ADENOMATOUS POLYPOSIS WITH EXTRACOLONIC MANIFESTATIONS (GARDNERS-SYNDROME). American journal of ophthalmology. 1990;110(5):550-61.

8.	Heyen F, Jagelman DG, Romania A, Zakov ZN, Lavery IC, Fazio VW, et al. PREDICTIVE VALUE OF CONGENITAL HYPERTROPHY OF THE RETINAL-PIGMENT EPITHELIUM AS A CLINICAL MARKER FOR FAMILIAL ADENOMATOUS POLYPOSIS. DISEASES OF THE COLON & RECTUM. 1990;33(12):1003-8.

9.	Burn J, Chapman P, Delhanty J, Wood C, Lalloo F, Cachon-Gonzalez MB, et al. The UK Northern region genetic register for familial adenomatous polyposis coli: use of age of onset, congenital hypertrophy of the retinal pigment epithelium, and DNA markers in risk calculations. Journal of medical genetics. 1991;28(5):289-96.

10.	Giardiello FM, Offerhaus GJ, Traboulsi EI, Graybeal JC, Maumenee IH, Krush AJ, et al. Value of combined phenotypic markers in identifying inheritance of familial adenomatous polyposis. Gut. 1991;32(10):1170-4.

11.	Houlston RS, Fallon T, Harocopos C, Williams CB, Davey C, Slack J. Congenital hypertrophy of retinal pigment epithelium in patients with colonic polyps associated with cancer family syndrome. Clinical genetics. 1992;42(1):16-8.

12.	Moore AT, Maher ER, Koch DJ, Charles SJ. Incidence and significance of congenital hypertrophy of the retinal pigment epithelium (CHRPE) in familial adenomatous polyposis coli (FAPC). Ophthalmic paediatrics and genetics. 1992;13(2):67-71.

13.	Shields JA, Shields CL, Shah PG, Pastore DJ, Imperiale SM, Jr. Lack of association among typical congenital hypertrophy of the retinal pigment epithelium, adenomatous polyposis, and Gardner syndrome. Ophthalmology. 1992;99(11):1709-13.

14.	Morton DG, Gibson J, Macdonald F, Brown R, Haydon J, Cullen R, et al. Role of congenital hypertrophy of the retinal pigment epithelium in the predictive diagnosis of familial adenomatous polyposis. The British journal of surgery. 1992;79(7):689-93.

15.	Hickey-Dwyer MU, Willoughby CE. Assessment of the value of congenital hypertrophy of the retinal pigment epithelium as an ocular marker for familial adenomatous polyposis coli. Eye (London, England). 1993;7 ( Pt 4):562-4.

16.	Bertario L, Bandello F, Rossetti C, Sala P, Fortini E, Spinelli P, et al. Congenital hypertrophy of retinal pigment epithelium (CHRPE) as a marker for familial adenomatous polyposis (FAP). European journal of cancer prevention : the official journal of the European Cancer Prevention Organisation (ECP). 1993;2(1):69-75.

17.	Maher ER, Barton DE, Slatter R, Koch DJ, Jones MH, Nagase H, et al. EVALUATION OF MOLECULAR-GENETIC DIAGNOSIS IN THE MANAGEMENT OF FAMILIAL ADENOMATOUS POLYPOSIS-COLI - A POPULATION-BASED STUDY. Journal of medical genetics. 1993;30(8):675-8.

18.	Hunt LM, Robinson MHE, Hugkulstone CE, Clarke B, Vernon SA, Gregson RHS, et al. CONGENITAL HYPERTROPHY OF THE RETINAL-PIGMENT EPITHELIUM AND MANDIBULAR OSTEOMATA AS MARKERS IN FAMILIAL COLORECTAL-CANCER. British journal of cancer. 1994;70(1):173-6.

19.	Santos A, Morales L, Hernandezquintela E, Jimenezsierra JM, Villalobos JJ, Panduro A. CONGENITAL HYPERTROPHY OF THE RETINAL-PIGMENT EPITHELIUM ASSOCIATED WITH FAMILIAL ADENOMATOUS POLYPOSIS. RETINA-THE JOURNAL OF RETINAL AND VITREOUS DISEASES. 1994;14(1):6-9.

20.	Hodgson SV, Bishop DT, Jay B. GENETIC-HETEROGENEITY OF CONGENITAL HYPERTROPHY OF THE RETINAL-PIGMENT EPITHELIUM (CHRPE) IN FAMILIES WITH FAMILIAL ADENOMATOUS POLYPOSIS. Journal of medical genetics. 1994;31(1):55-8.

21.	Campbell WJ, Spence RAJ, Parks TG. THE ROLE OF CONGENITAL HYPERTROPHY OF THE RETINAL-PIGMENT EPITHELIUM IN SCREENING FOR FAMILIAL ADENOMATOUS POLYPOSIS. International journal of colorectal disease. 1994;9(4):191-6.

22.	Depietri S, Sassatelli R, Roncucci L, Bertoni G, Landi P, Sabadini G, et al. CLINICAL AND BIOLOGIC FEATURES OF ADENOMATOSIS-COLI IN NORTHERN ITALY. Scandinavian journal of gastroenterology. 1995;30(8):771-9.

23.	Grobbelaar JJ, Ziskind A, de Jong G, Oosthuizen CJ, Kotze MJ. A novel deletion at codon 441 of the APC gene associated with ophthalmic lesions (CHRPE) in a South African family. Journal of medical genetics. 1996;33(5):384-6.

24.	Rossato M, Rigotti M, Grazia M, Turco AE, Bonomi L. Congenital hypertrophy of the retinal pigment epithelium (CHRPE) and familial adenomatous polyposis (FAP). Acta ophthalmologica Scandinavica. 1996;74(4):338-42.

25.	Valanzano R, Cama A, Volpe R, Curia MC, Mencucci R, Palmirotta R, et al. Congenital hypertrophy of the retinal pigment epithelium in familial adenomatous polyposis. Novel criteria of assessment and correlations with constitutional adenomatous polyposis coli gene mutations. Cancer. 1996;78(11):2400-10.

26.	Olea JL, Mateos JM, Llompart A, Obrador A. Frequency of congenital hypertrophy of the retinal pigment epithelium in familial adenomatous polyposis. Acta ophthalmologica Scandinavica. 1996;74(1):48-50.

27.	Traboulsi EI, Apostolides J, Giardiello FM, Krush AJ, Booker SV, Hamilton SR, et al. Pigmented ocular fundus lesions and APC mutations in familial adenomatous polyposis. Ophthalmic genetics. 1996;17(4):167-74.

28.	Tiret A, Taiel-Sartral M, Tiret E, Laroche L. Diagnostic value of fundus examination in familial adenomatous polyposis. The British journal of ophthalmology. 1997;81(9):755-8.

29.	Reck AC, Bunyan D, Eccles D, Humphry R. The presence of congenital hypertrophy of the retinal pigment epithelium in a subgroup of patients with adenomatous polyposis coli mutations. Eye (London, England). 1997;11 ( Pt 3):298-300.

30.	Ruhswurm I, Zehetmayer M, Dejaco C, Wolf B, Karner-Hanusch J. Ophthalmic and genetic screening in pedigrees with familial adenomatous polyposis. American journal of ophthalmology. 1998;125(5):680-6.

31.	Gebert JF, Dupon C, Kadmon M, Hahn M, Herfarth C, von Knebel Doeberitz M, et al. Combined molecular and clinical approaches for the identification of families with familial adenomatous polyposis coli. Annals of surgery. 1999;229(3):350-61.

32.	Cao X, Eu KW, Seow-Choen F, Zao Y, Cheah PY. APC mutation and phenotypic spectrum of Singapore familial adenomatous polyposis patients. European journal of human genetics : EJHG. 2000;8(1):42-8.

33.	Pang CP, Keung JW, Tang NL, Fan DS, Lau JW, Lam DS. Congenital hypertrophy of the retinal pigment epithelium and APC mutations in two Chinese families with familial adenomatous polyposis. Eye (London, England). 2000;14 ( Pt 1):18-22.

34.	Cetta F, Montalto G, Gori M, Curia MC, Cama A, Olschwang S. Germline mutations of the APC gene in patients with familial adenomatous polyposis-associated thyroid carcinoma: results from a European cooperative study. The Journal of clinical endocrinology and metabolism. 2000;85(1):286-92.

35.	Chen CS, Phillips KD, Grist S, Bennet G, Craig JE, Muecke JS, et al. Congenital hypertrophy of the retinal pigment epithelium (CHRPE) in familial colorectal cancer. Familial cancer. 2006;5(4):397-404.

36.	Cai SR, Zhang SZ, Zheng S. Clinical features of familial adenomas polyps in Chinese and establishment of its immortal lymphocyte cell lines. World journal of gastroenterology. 2007;13(20):2858-61.

37.	Garzón-Benavides M, Pizarro-Moreno A, García-Lozano R, Herrero-Garrido MI, Hervás-Molina AJ, Márquez-Galán JL, et al. Andalusian Registry for familial adenomatous polyposis. Analysis of patients included. Revista espanola de enfermedades digestivas. 2010;102(11):653-7.

38.	Burger B, Cattani N, Trueb S, de Lorenzo R, Albertini M, Bontognali E, et al. Prevalence of skin lesions in familial adenomatous polyposis: a marker for presymptomatic diagnosis? The oncologist. 2011;16(12):1698-705.

39.	Nusliha A, Dalpatadu U, Amarasinghe B, Chandrasinghe PC, Deen KI. Congenital hypertrophy of retinal pigment epithelium (CHRPE) in patients with familial adenomatous polyposis (FAP); a polyposis registry experience. BMC research notes. 2014;7:734.

40.	Mirinezhad SK, Mousavi F, Baghri M, Sepehri B, Ghavidel A, Ghojazadeh M, et al. Congenital Hypertrophy of Retinal Pigment Epithelium for Diagnosis of Familial Adenomatous Polyposis - the First FAP registry in Iran. Asian Pacific journal of cancer prevention : APJCP. 2018;19(1):167-9.

41.	de Oliveira JC, Viana DV, Zanardo C, Santos EMM, de Paula AE, Palmero EI, et al. Genotype-phenotype correlation in 99 familial adenomatous polyposis patients: A prospective prevention protocol. Cancer medicine. 2019;8(5):2114-22.

42.	Cai S, Yu Y, Xie X, Huang Y, Song Y, Zhan S, et al. Study of diagnostic value of congenital hypertrophy of retinal pigment epithelium in Chinese familial adenomatous polyposis patients. European journal of cancer prevention : the official journal of the European Cancer Prevention Organisation (ECP). 2022;31(5):422-9.

43.	Carvalho AA, Crespo TS, Násser LS, Maia CMF, Fonseca CAD, Silveira CM, et al. Use of congenital hypertrophy of the retinal pigment epithelium as a clinical sign of familial adenomatous polyposis. Arquivos brasileiros de oftalmologia. 2024;88(3).
